# Exosome-mediated breast cancer chemoresistance via miR-155 transfer

**DOI:** 10.1038/s41598-018-19339-5

**Published:** 2018-01-16

**Authors:** Juliana Carvalho Santos, Natália da Silva Lima, Luis Otavio Sarian, Ander Matheu, Marcelo Lima Ribeiro, Sophie Françoise Mauricette Derchain

**Affiliations:** 1 Women’s Health Hospital “Prof Dr José Aristodemo Pinotti” (CAISM), State University of Campinas (UNICAMP), Campinas, SP Brazil; 2 Clinical Pharmacology and Gastroenterology Unit, São Francisco University, São Francisco University, Bragança Paulista, SP Brazil; 3 Cellular Oncology Group, Biodonostia Health Research Institute, San Sebastian, Spain

## Abstract

Breast cancer remains the most prevalent cause of cancer mortality in woman worldwide due to the metastatic process and therapy resistance. Resistance against cancer therapy is partially attributed to cancer stem cells (CSCs). These cells arise from epithelial cells undergoing epithelial-to-mesenchymal transition (EMT) and might be responsible for tumor recurrence. In this study, we reported the relevance of miR-155 upregulation in chemoresistant cells associated with EMT. Notably, we found miR-155 induction in exosomes isolated from CSCs and resistant cells, followed by resistant cells’ exosome transfer to the recipient sensitive cells. Functionally, miR-155 mimic assay showed an enrichment in miR-155 from exosome concomitant with miR-155 exosome transfer to breast cancer cells. In parallel to these effects, we also observed EMT change in miR-155 transfected cells. The chemoresistance phenotype transfer to sensitive cells and the migration capability was analyzed by MTT and scratch assays and our results suggest that exosomes may intermediate resistance and migration capacity to sensitive cells partly through exosome transfer of miR-155. Taken together, our findings establish the significance of exosome-mediate miR-155 chemoresistance in breast cancer cells, with implications for targeting miR-155 signaling as a possible therapeutic strategy.

## Introduction

Despite significant advances in chemotherapy, several studies have shown that resistance caused by repetitive and long-term drug administration during treatment remains the major factor for treatment failure and death in breast cancer patients^1^. The chemoresistance acquisition requires multiple regulatory changes of tumor microenvironment, which is composed partially by exosomes. Exosomes are small vesicles (50–150 nm) that contain mRNAs, miRNAs (miRs), and proteins, and are released from diverse cell types, including cancer cells and cancer stem cells (CSCs), allowing intercellular communication^2^.

Breast cancer is the most common type of tumor worldwide among women. The resistance against cancer therapy is attributed partially to CSCs. These cells are recognized as having self-renewal ability, high expression of specific surface cell markers (CD44 and ALDH1), low expression of CD24, and are responsible for tumor recurrence and metastasis^3^. The CSCs can arise from epithelial cells undergoing epithelial-to-mesenchymal transition (EMT), a process characterized by loss of E-CADHERIN (E-CAD) expression, through transcriptional repressors such as SNAIL and SLUG. These events are accompanied by an increase of stemness-related transcription factors, BMI1 and EZH2, which may trigger the transformation of epithelial cells into mesenchymal state with the ability to invade other tissues^4,5^. Therefore, identifying the drug resistance mechanisms of CSCs is crucial to understand and determine therapeutic targets most suitable for breast cancer.

Current studies provide strong evidence that miRs, small non-coding RNAs that control gene expression, have also been associated with CSCs, EMT and drug resistance^6^. Some miRs carried by exosomes from breast cancer cells^7^, as well as circulating exosome-miRs from plasma of patient-derived xenograft (PDX) mice and breast cancer patients^8^, are differently expressed from those secreted by normal breast cells, which suggests a potential use of exosomes-miRs as biomarkers for breast cancer diagnosis. Among the miRs, miR-155 is an oncomiR that is overexpressed in several cancers^9^. A growing number of studies highlights the role of miR-155 in breast cancer drug resistance development^10,11^. Interestingly, miR-155 mediates the loss of C/EBP-β activity and is closely involved with TGF-β-induced EMT, invasion, and metastasis^12^. Moreover, miR-155 targets directly FOXO-3a 3′-UTR downregulating its expression to regulate the drug response of breast cancer cells^13^.

Tumors comprise a heterogeneous population of cells, those that will be attacked and eliminated by chemotherapy - the sensitive ones, and those that will survive the treatment, named drug-resistant cells. The resistant-cell population may be able to spread the resistance features to residual cells. Previous studies showed that chemoresistant cells are enriched in exosomes that may act as genetic modulators^14,15^. Although exosomes have been increasingly researched, the mechanisms underlying chemoresistance remains elusive. To expand this knowledge, we investigate the EMT-mediated chemoresistance transfer through miR-155 exosomes delivery.

## Results

### Chemosensitivity response

**Figure 1:**
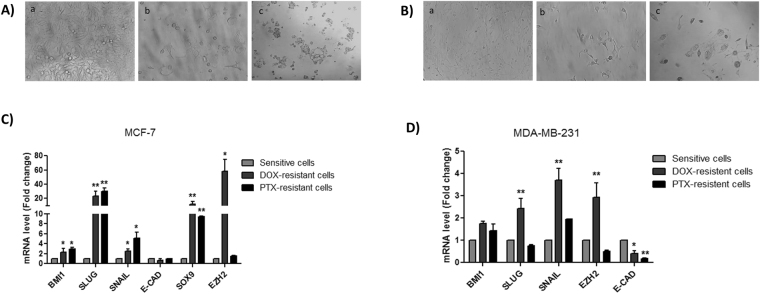
Morphological and molecular changes of breast cells in response to doxorubicin and paclitaxel treatment. (**A**) MCF-7 morphological changes after chemoresistance induction; (**B**) MDA-MB-231 morphological changes after chemoresistance induction, a – sensitive cells, b – DOX-resistant cells, c – PTX-resistant cells; (**C**) EMT markers expression in sensitive, DOX and PTX-resistant MCF-7 cell line; (**D**) EMT markers expression in sensitive, DOX and PTX-resistant MDA-MB-231 cell line. The results were analyzed using t-test. *p < 0.05 and **p < 0.01 compared with the sensitive cells group.

Recent evidence indicated that EMT inhibition does not impair the ability of breast tumor cells to form lung metastasis, but it is involved in the metastatic process in women exposed to chemotherapy^16^. The acquisition of EMT process has been linked with disease aggressiveness, which may have been caused by stemness properties acquisition and resistance to standard therapies, which include anthracyclines and taxanes. To determine chemosensitivity of MCF-7 and MDA-MB-231 cell lines to Doxorubicin (DOX) and Paclitaxel (PTX), the cell lines were treated with stepwise drug concentrations. The cell viability was examined using MTT assay and IC50 was calculated and used to induce chemoresistance (Table 1). After chemoresistance induction, we observed a morphological change which suggests EMT acquisition (Fig. 1A and B). Indeed, we found higher mRNA levels of *BMI1*, *SLUG*, *SNAIL*, *SOX9* and *EZH2*, and lower mRNA level of *E-CAD* in resistant cells when compared to sensitive cells (Fig. 1C and D), confirming EMT molecular changes.

**Table 1:** Chemosensitivity to Doxorubicin and Paclitaxel in MCF-7 and MDA-MB-231 cell lines.

Cell line	Doxorubicin IC50 (nM)	Paclitaxel IC50 (nM)
MCF-7	320	60
MDA-MB-231	320	40

### miR-155 is upregulated in breast CSCs and in chemoresistant cells

**Figure 2:**
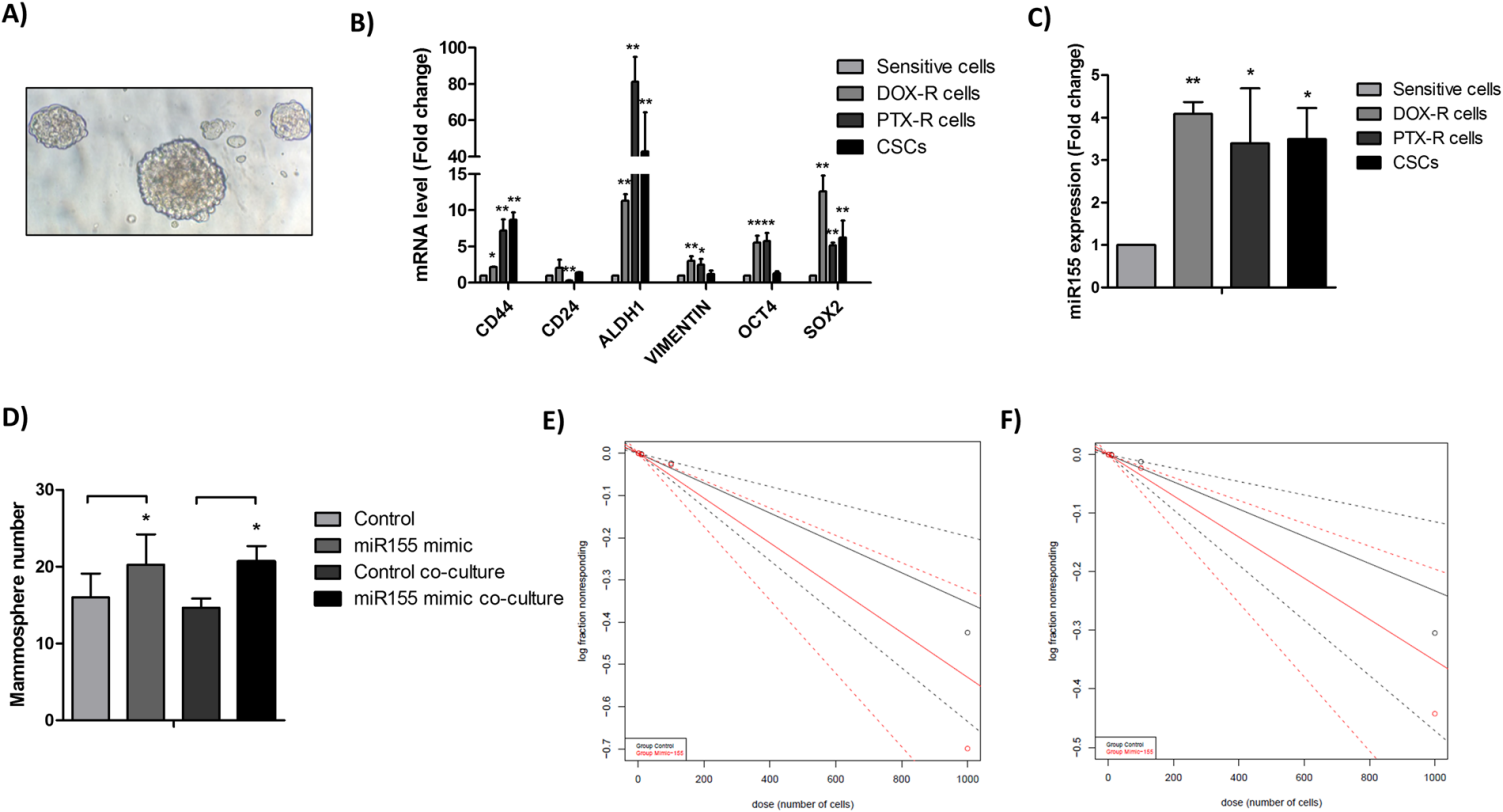
Breast CSC population characteristics. (**A**) 3D morphological characteristic of mammospheres generated from MCF-7 cell line; (**B**) Breast CSCs markers in mammospheres (CSCs) and chemoresistant breast cells; (**C**) miR-155 expression in mammospheres (CSCs) and chemoresistant breast cells; (**D**) Mammosphere number following miR-155 transfection or control and co-culture with exosomes from MCF-7 cells transfected with miR-155 or control; The results were analyzed using t-test. *p < 0.05 and **p < 0.01 compared with the sensitive cells or control group; (**E**) Mammosphere formation from miR-155 transfection or control using the ELDA platform; (**F**) Mammosphere formation from cells co-cultured with exosomes from MCF-7 cells transfected with miR-155 or control using the ELDA platform. The slope of the line is the log-active cell fraction. Solid lines depict the mean, the dotted lines give the 95% confidence interval, and circles indicate the values obtained in each cell dilution.

Oncospheres are correlated with CSC behavior and are broadly used for identifying CSCs *in vitro*^17^. It has been described that miR-155 is an important EMT and CSCs regulator^18^. To determine this relation, we generated mammospheres from MCF-7 cell line (Fig. 2A) and we found that *CD44*, *ALDH1*, *VIMENTIN*, *OCT4* and *SOX2* breast CSCs markers were higher in the CSCs and in chemoresistant cells than in parental cells (Fig. 2B). Likewise, our data showed a miR-155 upregulation in both, CSCs and chemoresistant cells (Fig. 2C). It is often suggested that breast CSCs are resistant to conventional chemotherapy. When cells overexpressing miR-155 were used to form the mammospheres, an increase in mammosphere formation was observed (Fig. 2D,E and F), indicating that miR-155 is critical for breast CSCs formation. Together, these data suggest that miR-155 upregulation significantly increases the population of stem-like CSCs among breast cancer cells and may be involved in chemoresistance process.

### Exosomes secreted from breast CSCs and chemoresistant cells present miR-155 enrichment

**Figure 3:**
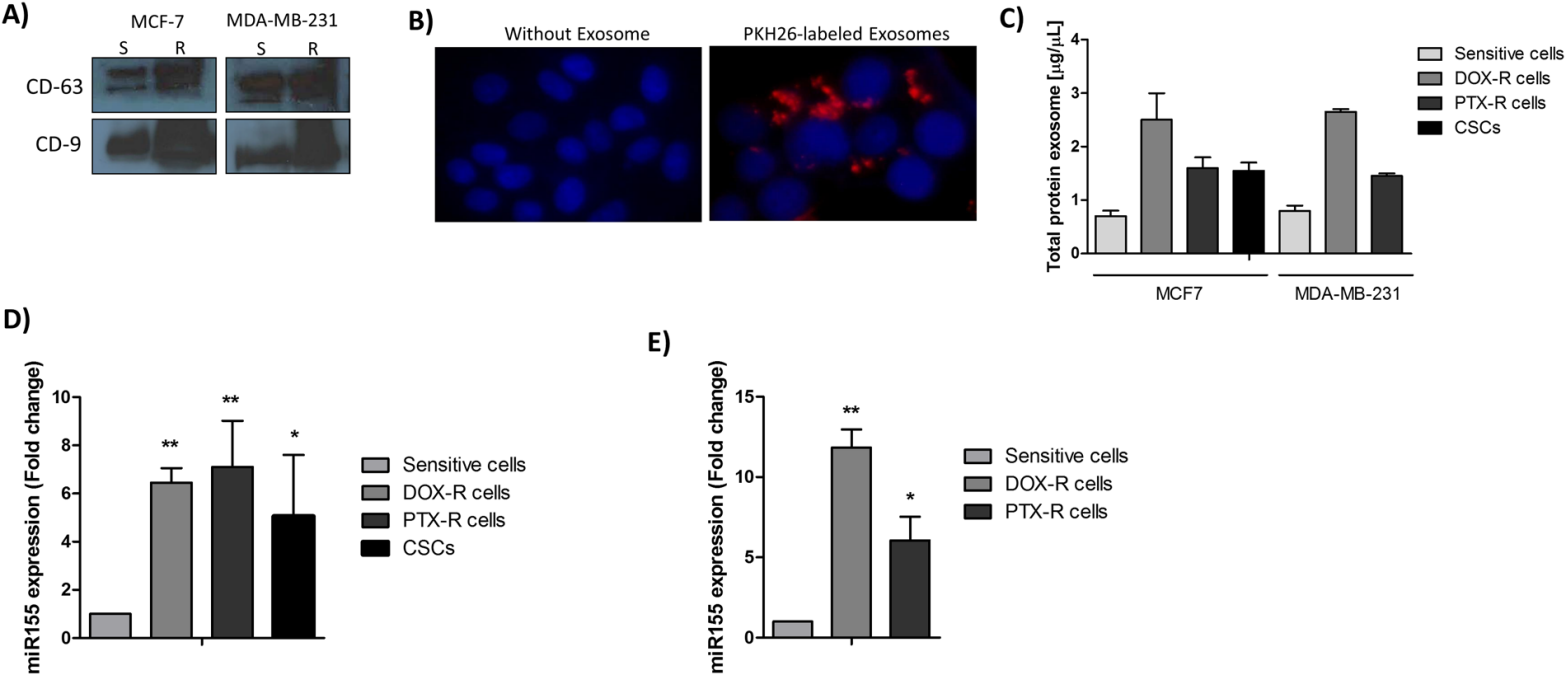
Characterization of exosomes. (**A**) Western blot for the exosome-related proteins CD-63 and CD-9, S – exosomes from sensitive cells/R – exosomes from resistant cells; In the figure are reported the cropped blots, and full-length blots are presented in Supplementary Figure S1; (**B**) Representative fluorescence microscopy showing uptake of PKH26-labeled exosomes into recipient MCF-7 cells, blue – nuclei, red - PKH26-labeled exosomes; **(C)** Exosome protein concentrations from mammospheres (CSCs), sensitive, DOX and PTX resistant MCF-7 and MDA-MB-231 breast cells; (**D**) miR-155 level into exosomes from sensitive, DOX-resistant, PTX-resistant and CSCs MCF-7 cells; (**E**) miR-155 level into exosomes from sensitive, DOX-resistant, and PTX-resistant MDA-MB-231 cells. The results were analyzed using t-test. *p < 0.05 and **p < 0.01 compared with the sensitive cells group.

Cancer progression during the treatment is dependent on cross-talk between tumor chemoresistant cells and the microenvironment, which is enriched in exosomes. Based on current finding that miR-155 may be involved with EMT, and considering the close intercommunication with adjacent cells that may take an active part in the transmission of drug resistance, we analyzed miR-155 expression levels in exosomes from CSCs, sensitive, and chemoresistant cells. First of all, western blotting for the exosome-related proteins, CD-63 and CD-9 revealed that the exosomes were positive for both exosome markers (Fig. 3A), confirming an enrichment in exosomes. Then, we examined whether the exosomes from donor cells could be transferred to recipient cells. When we labeled the MCF-7 cell–derived exosomes with PKH26 (red fluorescence dye), a red staining on the cell was observed, suggesting the exosome incorporation (Fig. 3B). Interestingly, exosomes from CSCs and DOX and PTX-resistant cells expressed higher amounts of protein than the sensitive ones (Fig. 3C). In addition, we detected an increase of miR-155 in CSCs and resistant cells’ exosomes (Fig. 3D and E). This result indicates that exosomes could potentially spread chemoresistance to other cells.

### Exosomes transfer alters EMT properties and chemoresponse

**Figure 4:**
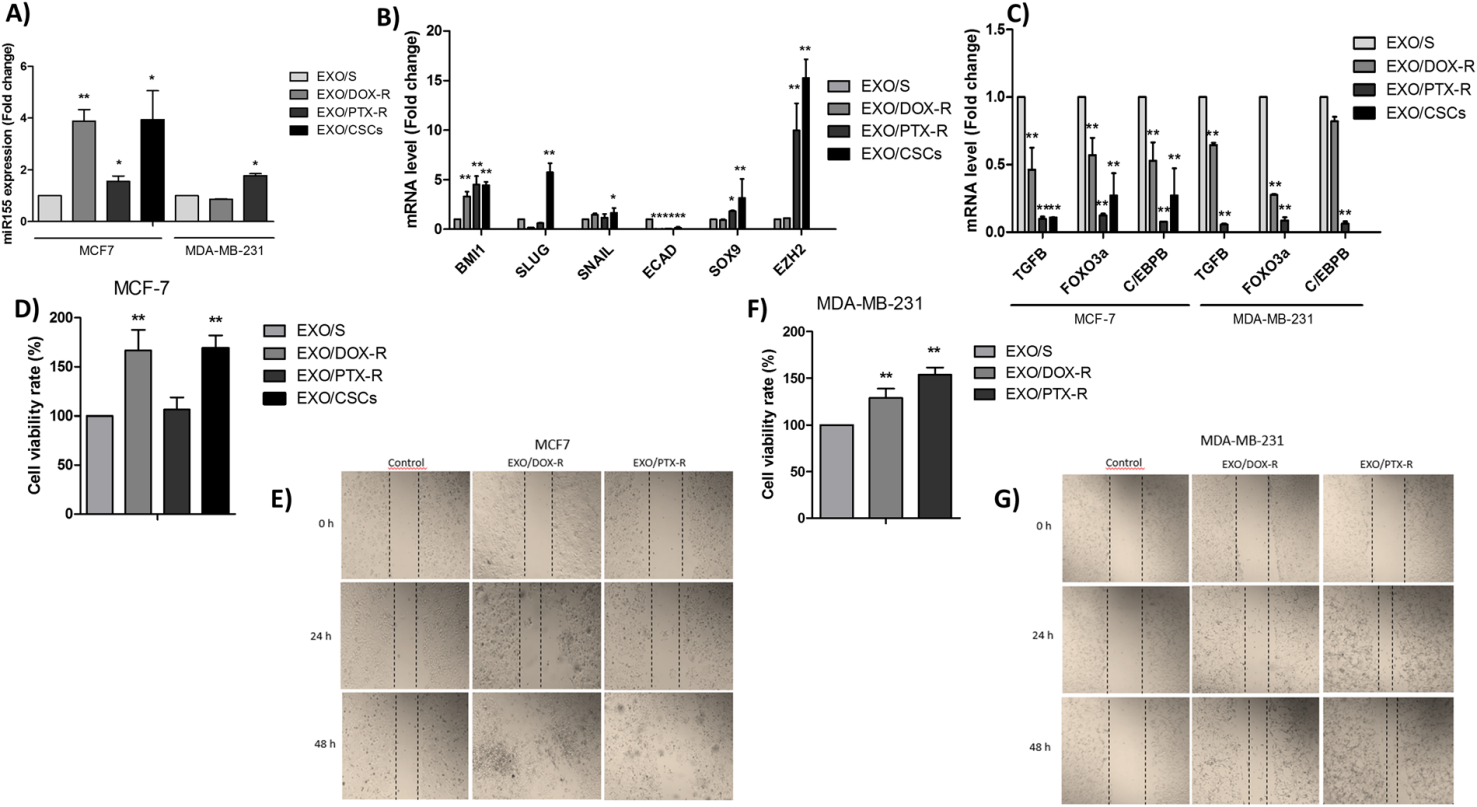
Effects of exosome co-cultures. (**A**) miR-155 level in MCF-7 cells following co-culture with exosomes from sensitive, DOX-resitant, PTX-resistant and CSCs MCF-7 cells, and miR-155 level in MDA-MB-231 following co-culture with exosomes from sensitive, DOX-resistant and PTX-resistant MDA-MB-231 cells; (**B**) mRNA levels of EMT markers following co-culture with exosomes from DOX-resitant, PTX-resistant and CSCs breast cells; (**C**) miR-155 targets expression following co-culture with exosomes from DOX-resitant, PTX-resistant and CSCs breast cells in MCF-7 and MDA-MB-231 cells; (**D**) Chemosensitivity to doxorubicin and paclitaxel following co-culture with exosomes from sensitive, DOX and PTX resistant cells, and mammospheres (CSCs) MCF-7 cells; (**E**) Migration assay following co-culture with exosomes from sensitive, DOX and PTX resistant MCF-7 cells; (**F**) Chemosensitivity to doxorubicin and paclitaxel following co-culture with exosomes from sensitive, DOX and PTX-resistant MDA-MB-231 cells; (**G**) Migration assay following co-culture with exosomes from sensitive, DOX and PTX resistant MDA-MB-231 cells. The results were analyzed using t-test. *p < 0.05 and **p < 0.01 compared with the EXO/S group.

To further extend the effect of exosomes cross-talk, we tested whether CSCs, DOX- and PTX-resistant cells could alter the EMT process and chemoresponse in breast cancer sensitive cells through exosome incorporation. Our data show an increase in miR-155 expression in cells co-cultured with exosomes from CSCs and chemoresistant cells (Fig. 4A). In addition, we found a gain of EMT markers *BMI1*, *SLUG*, *SNAIL*, *SOX9* and *EZH2* concomitant with *E-CAD* and miR-155 targets TGF-β, FOXO-3a and C/EBP-β mRNA loss in sensitive cells co-cultured with exosomes from CSCs and chemoresistant cells (Fig. 4B and C).

Transmitted chemoresistance was subsequently assessed in sensitive cells co-cultured with exosomes from CSCs and chemoresistant cells and treated with IC50 for cell viability determination by MTT assay. As seen in Fig. 4D, the MCF-7 cells were 60% more resistant to doxorubicin when they received exosomes from DOX-resistant and CSCs cells. MDA-MB-231 cells were 29% and 53% more resistant to doxorubicin and paclitaxel when they received exosomes from DOX- and PTX-resistant cells, respectively, when compared with cells that received exosomes from sensitive cells (Fig. 4F), further indicating that drug-resistance transfer should be ascribed to exosomes. The migration capability of breast cancer cells after co-culture with exosomes from sensitive, DOX and PTX-resistant cells was assessed using scratch assay. The migration rate of MCF-7 (Fig. 4E) and MDA-MB-231 (Fig. 4G) cells was higher when they received exosomes from respective resistant cells than when they received exosomes from sensitive ones, indicating higher migration potential promoted by exosomes.

### Increased miR-155 expression increases miR-155 content of exosomes, leading to EMT–associated chemoresistance

**Figure 5:**
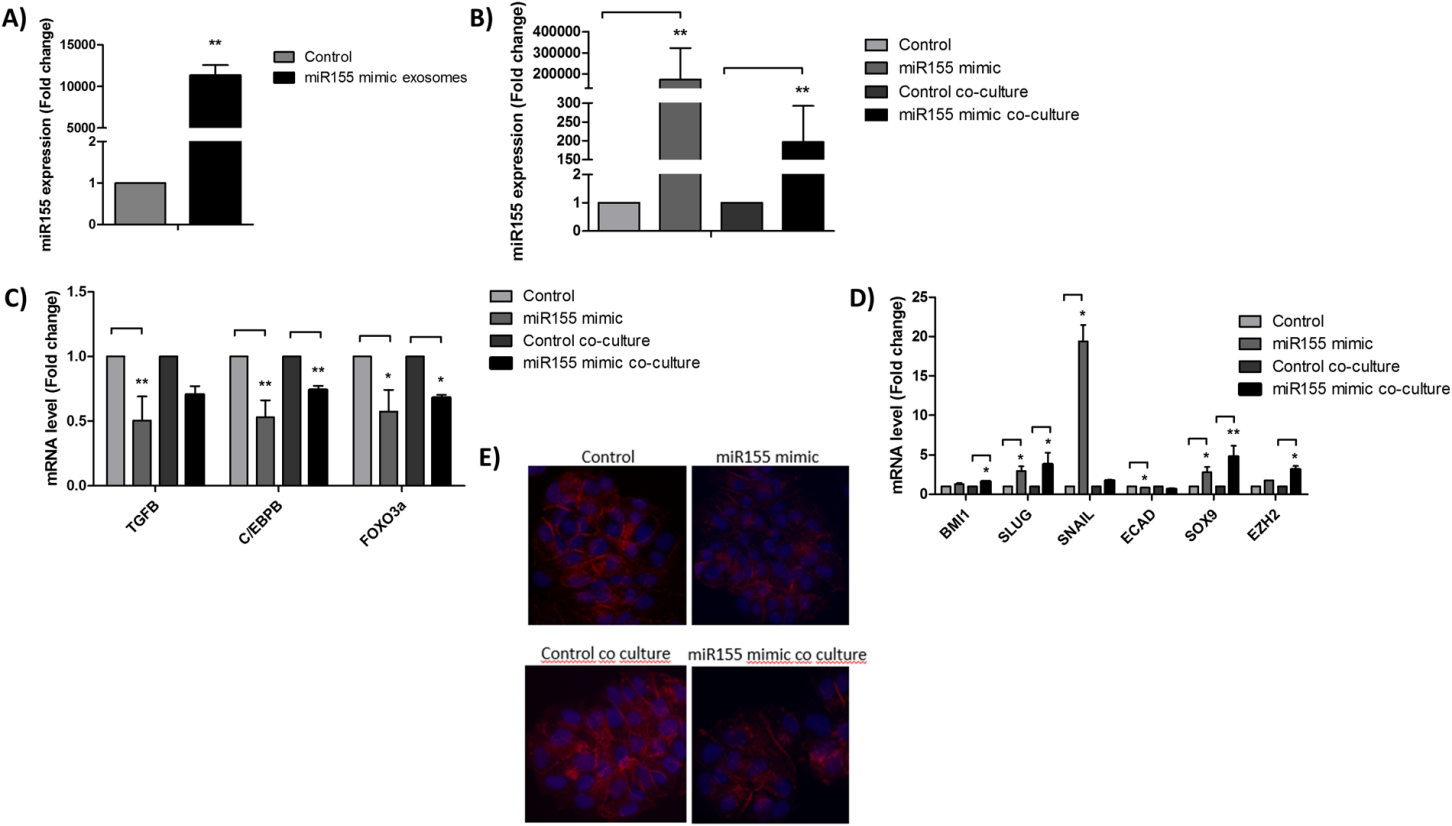
miR-155 transfection. (**A**) miR-155 level into exosomes from MCF-7 cells transfected with miR-155 (mimic) or negative control; (**B**) miR-155 expression in MCF-7 cells transfected with miR-155 or control and MCF-7 cells co-cultured with exosomes from cells transfected with miR-155 or control; (**C**) miR-155 targets expression in MCF-7 cells transfected with miR-155 or control and MCF-7 cells co-cultured with exosomes from cells transfected with miR-155 or control; (**D**) EMT mRNA markers expression in MCF-7 cells transfected with miR-155 or control and MCF-7 cells co-cultured with exosomes from cells transfected with miR-155 or control. The results were analyzed using t-test. *p < 0.05 and **p < 0.01 compared with the control group. (**E**) E-cadherin immunofluorescence in MCF-7 cells transfected with miR-155 or control and MCF-7 cells co-cultured with exosomes from cells transfected with miR-155 or control.

To investigate whether chemoresistance could be assigned to miR-155-exosome delivery, exosomes were isolated from MCF-7 cells transfected with miR-155 (mimic) or negative control and co-cultured with MCF-7 cells. The results showed that miR-155 transfection drastically increased the level of miR-155 into exosomes (Fig. 5A). Furthermore, the co-culture with exosomes derived from cells overexpressing miR-155, strongly induced miR-155 level in recipient cells (Fig. 5B) suggesting that miR-155 is transferred by exosomes.

Its well-known that miR-155 mediates the loss of C/EBP-β, which in turn, causes loss of TGF-β and leads to EMT in breast cancer cells^19^. Additionally, FOXO3a, which is a validated miR-155 target, may affect the drug susceptibility thought it’s downregulation in breast cancer cells^19^. In accordance with this, we observed a repression of TGF-β, C/EBP-β and FOXO3a in cells transfected with miR-155 as well as in cells that received exosomes from cells overexpressing miR-155 (Fig. 5C). Interestingly, both cells caused EMT marker induction and repression of E-cadherin (Fig. 5D and E), confirming EMT acquisition through miR-155 exosome transfer.

**Figure 6:**
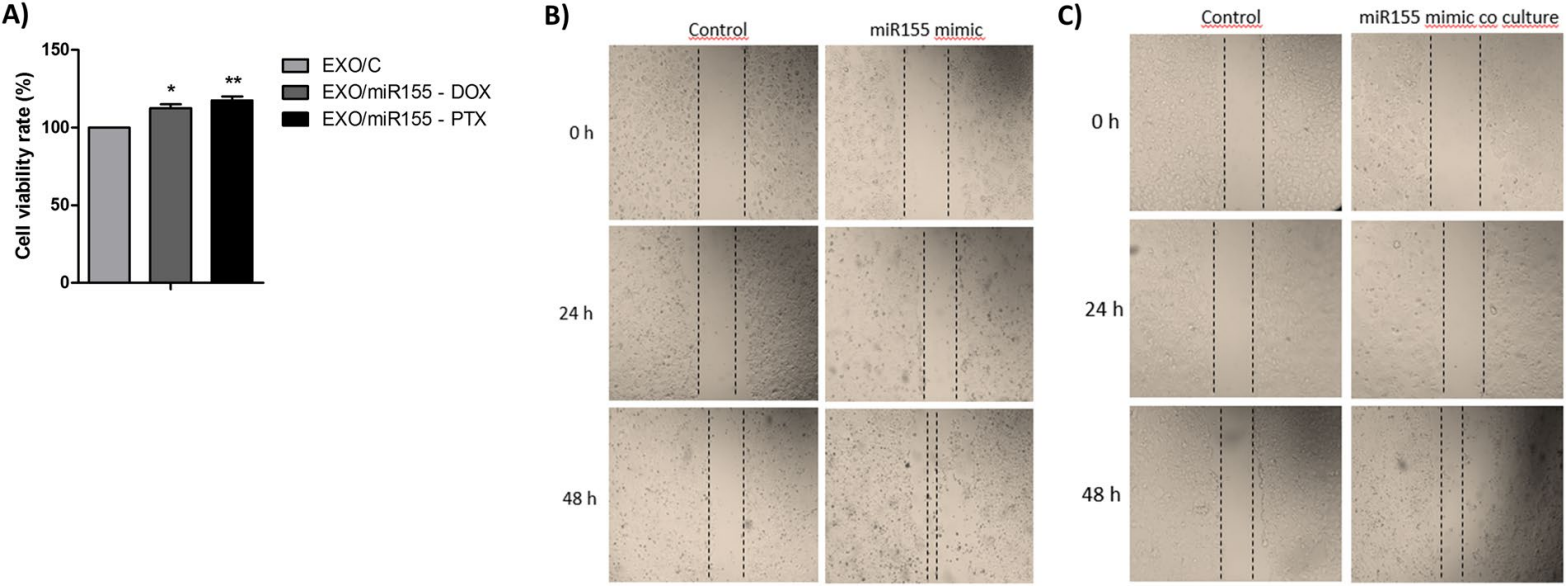
Chemosensitivity and migration capacity of cells co-cultured with exosomes from cells transfected with miR-155. (**A**) Chemosensitivity to doxorubicin and paclitaxel following co-culture with exosomes from MCF-7 cells transfected with miR-155; (**B**) Migration assay of MCF-7 cells transfected with miR-155 or control; (**C**) Migration assay of MCF-7 cells co-cultured with exosomes from cells transfected with miR-155 or control. The results were analyzed using t-test. *p < 0.05 and **p < 0.01 compared with the EXO/C group.

To assess the role of exosomes’ miR-155 in chemoresistance, we assessed the chemoresponse in breast cancer cells co-cultured with exosomes from miR-155 mimic or control cells. The exosomes from miR-155 mimic cells were able to significantly induce DOX and PTX resistance in breast cells when compared to the exosomes from control cells (Fig. 6A). Furthermore, the cells with miR-155 induction (Fig. 6B) and the cells that received exosomes from miR-155 inducted (Fig. 6C) also caused higher migration rate. These findings indicate that the chemoresistance and the migration ability can be promoted by miR-155 through exosomes transfer.

## Discussion

Exosomes have been shown to contribute to cancer progression and chemoresistance in a variety of cancers^20,21^. Chemoresistance is a major barrier to the successful treatment of breast cancer, and to clarify the resistance-associated mechanisms is urgently needed. The exosomes present in tumor microenvironment can bind and be internalized by the neighbor cells which modify recipient cell phenotypes through exchange of genetic information. Importantly, the mRNA carried by exosomes was shown to be translated into protein in recipient cells, supporting the functional nature of exosomes^22^. Interestingly, Valadi *et al*.^22^ have found 121 miRs in exosomes from mast cells and showed that some miRs are uniquely packed into exosomes. The current study expands the understanding into the putative involvement of DOX and PTX resistance breast cancer cells and miR-155 overexpression through breast CSCs and chemoresistant cells’ exosome secretion.

Doxorubicin and paclitaxel are two agents commonly used in patients with breast cancer, and were used to better understand the chemoresistance mechanism in current study. MCF-7 (estrogen receptor-positive) and MDA-MB-231 (estrogen receptor-negative) DOX and PTX resistant cells, and the paired sensitive cells were therefore chosen as the subjects in this study. The choice of these cell lines was based on its differential molecular features and drug response^23^, considering that the investigation of drug resistance mechanisms are extremely important to personalized medicine, since specific anti-breast cancer agents act in a different way, depending of tumor subtypes.

Since CSCs resist to treatment and allow to tumor regrowth post-treatment^24^, we also have included mammospheres generated from MCF-7 cells in this work. We observed changes in morphological properties concomitant with molecular EMT acquisition in breast cancer resistant cells.

Mammosphere culture mimics *in vitro* breast CSC population. It has been shown that these cells undergo EMT process, generating sub-population with CD44^+^/CD24^−^/ALDH1^+^ phenotype^25^. Indeed, there was higher mRNA CD44, ALDH1, VIMENTIN, OCT4 and SOX2 levels in CSCs and resistant cells compared to parental cells. These results showed a putative link between EMT and the gain of CSC-like properties as a cause of chemoresistance. Previous studies have demonstrated that the drug-resistant cancer cells possess features of EMT in different cancer types, including breast cancer^26,27^. Our results are also consistent with clinical findings in which the authors showed an increase of CSC markers in biopsies taken from patients undergoing 12-week chemotherapy regimens^28^.

Several reports have shown that miR-155 acts as an oncomiR in human cancers by targeting tumor suppressors genes^29,30^. It has been shown that miR-155 promotes EMT and CSCs phenotypes^18^, both contributing to drug resistance. Our results are in agreement with other studies showing that miR-155 overexpression is involved in breast cancer chemoresistance^13,10^, and also suggest that miR-155 might be involved in the acquisition of stemness properties in breast cancer cells. Likewise, Chiu *et al*.^31^ revealed *in vitro* and *in vivo* study that miR-155 is linked to lung cancer resistant phenotype through FOXO3a repression and an enhancement in CSC properties. Based on these findings, we suggest that upregulation of miR-155 in breast CSC and chemoresistant cells may be involved in the acquired stemness resistance mechanisms.

The emerging evidence that cells may communicate with each other through exosomes has recently highlighted the importance of these vesicles in resistance transmission and therapy failure^32^. To further extend this idea, we have cultured breast sensitive cells with exosomes secreted by breast CSCs and chemoresistant cells. It is well known that some tetraspanins, as CD-63 and CD-9, are exosome-specific markers and are commonly used for exosome characterization^33^. In the present work, the exosomes were CD-63 and CD-9 enriched indicating that the studied vesicles are exosomes. Furthermore, the exosome incorporation by breast cancer sensitive cells was confirmed by PKH26 staining indicating that exosomes could potentially mediate cell-to-cell communication. In addition, our experiments indicate that the exosomes from breast CSCs and chemoresistant cells significantly overexpressed miR-155, raising the possibility of breast cancer chemoresistance was due to high exosome miR-155 content.

Since that chemoresistant breast cancer cells are an abundant source of exosomes^14^, we analyzed whether internalized exosomes might influence the breast cells chemosensibility and migration ability. Our results from co-culture assays showed that there was an acquisition of EMT properties and migration capability with high miR-155 expression as well as less chemosensitivity when the cells received exosomes from chemoresistant cells. Our data are in agreement with previous studies reporting the enhance of exosome-induced migration^34,35^ and the ability of drug-resistant cells in communicating with sensitive cells and transmitting resistance via miR-exosomes delivery in breast cancer^15,32^, and also postulate that miR-155 might be involved. It has recently been described that anti-cancer drugs strongly increase tumor cell secretion of exosomes facilitating the chemoresistance and post-therapy relapse through signaling pathways activation and inflammation induction^36^. However, there are few studies demonstrating the chemosensitivity transmission by exosomes released from CSCs. In this sense, Ji *et al*.^37^ showed that exosomes from mesenchymal stem cells could confer multi-drug resistance in gastric cancer *in vitro* and *in vivo*. Additionally, Hu *et al*.^38^ showed that fibroblasts derived exosomes prime CSCs to become more chemoresistance via WNT signaling pathway.

To further investigate the miR-155 role in exosome chemoresistance transmission, we overexpressed miR-155 in breast cancer cells which led to high miR-155 exosome level. When we cultivated these exosomes with breast cancer cells, there was also a miR-155 enrichment in recipient cells, indicating miR-155 transfer through exosomes. It has been shown that, C/EBP-β (a mediator of TGF-β) and FOXO3a are miR-155-validated targets^39,40^. Moreover, TGF-β is a cytokine that plays central role in EMT, is also regulated by miR-155^41^. Indeed, the overexpression of miR-155 led to the downregulation of its target genes, in both miR-155 mimic and miR-155 mimic´ exosome co-cultured cells, confirming this regulatory effect. Despite some evidence showing that miR-155 can promotes EMT and CSC phenotypes^42,43^, how miR-155 regulates EMT remains unclear. In the current work, we show that breast cancer cells overexpressing miR-155 acquired EMT properties, promoted higher migration rate, and were able to form more mammospheres, which suggest that miR-155 may regulate certain genes related to breast CSCs that are resistant to therapy. Interestingly, this landscape was also observed when the exosomes expressing high miR-155 level were endocytosed into the breast cancer cells, indicating the functional role of miR-155 exosome delivery. Mikamori *et al*.^44^ have reported in pancreatic ductal adenocarcinoma model, that miR-155 induces exosome secretion that causes gemcitabine resistance. In accordance to this study, our data shows that the cells that received exosomes from overexpressing miR-155 cells were significantly more resistant to DOX and PTX than cells that received exosomes from control cells, which suggests that miR-155 effectively elicits PTX resistance in breast cells via exosome uptake.

**Figure 7:**
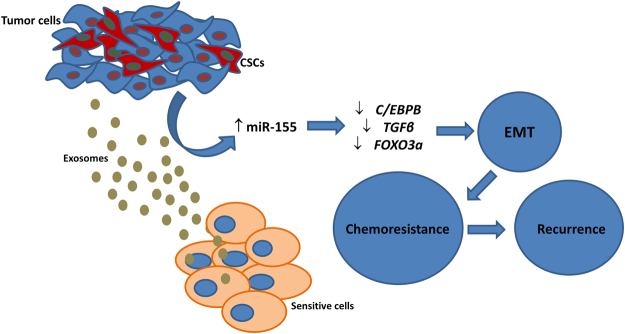
Putative mechanism of cell-to-cell communication exosome-mediated resistance transmission.

Altogether, our results support a putative mechanism of cell-to-cell communication miR-155 exosome-mediated resistance transmission (Fig. 7) and suggest that miR-155-targeting therapies combined with conventional chemotherapy might be useful to combat chemoresistance. Therefore, this study offers a potential target for miR-155 delivery through exosomes, and highlights the importance of inhibiting transfer of drug-resistance from resistant breast cells to sensitive ones, through exosomes, for success of current therapeutic approaches.

## Methods

### Cell lines culture

The human breast cancer cell lines MCF-7 and MDA-MB-231 were purchased from the Cell Bank Rio de Janeiro (Rio de Janeiro, RJ, Brazil). Both cell lines were cultured in DMEM medium (Life Technologies, Alemeda, CA, USA) supplemented with 10% FCS, 100 U/ml penicillin, and 100 mg/ml streptomycin at 37 °C under a water-saturated 95% air-5% CO_2_ atmosphere.

### miR-155 mimic transfections

MCF-7 cells were seeded one day before transfection. Next, cells were incubated with miR-155 mimic or controls for 6 h using the transfection reagent Lipofectamine 2000 (Invitrogen). About 30 nM of hsa-miR-155-5p or miR negative control (random sequences) were used for transfecting assays (Ambion, Grand Island, NY).

### Mammospheres formation

It is generally stated that breast CSCs can be isolated or enriched by culturing cells in non-adherent and non-differentiating conditions in order to form mammospheres^45,46^. The mammosphere serum-free culture model has been widely used to identify and enrich for breast CSCs from breast cancer cell lines and was used in current study. A hundred thousand cell per mL of MCF-7 cell line was seeded as cell suspensions into ultralow attachment plates and used to mammospheres formation (named CSCs). The cells were grown for 7 days in stem cell media - DMEM/F12 medium (Sigma-Aldrich, USA, MO) supplemented with 20 ng/mL of epidermal growth factor (EGF) and basic fibroblast growth factor (bFGF), 1 × N2 and 1 × B27 (Thermo Scientific, USA, IL), 100 U/mL penicillin, and 100 mg/mL streptomycin and were maintained at 37 °C under a water-saturated 95% air-5% CO_2_ atmosphere. One mL of fresh stem cell media was added to each plate every two days. The number of formed primary mammospheres with a diameter larger than 50 μm was counted under an inverted microscopy and harvested by centrifugation. The RNA from mammospheres was extracted for breast cancer stem cells markers analysis (CD44, CD24, ALDH1, VIMENTIN, OCT4 and SOX2).

### Limiting dilution assay

The *in vitro* limiting dilution assay is a useful method to determine the breast cancer initiating cell frequency of a mammosphere population. MCF-7 cells in following conditions - miR-155 mimic or control; co-cultered with exosomes from miR-155 mimic or control - were resuspended in stem cell media and serially diluted to obtain the final cell concentrations of 1000 cells/well (24 wells), 100 cells/well (96 wells), 10 cells/well (192 wells) and 1 cell/well (288 wells) of p96 plates. The cells were then incubated at 37 °C for at least 7 days and the mammosphere rate from each condition was used to calculate breast CSCs frequency, using the extreme limiting dilution analysis (ELDA) calculation (http://bioinf.wehi.edu.au/software/elda).

### Chemoresistance induction

The DOX-resistant and PTX-resistant variant of both MCF-7 and MDA-MB-231 cell lines were established by continuous culture in medium containing stepwise increasing concentration of drug and maintained in medium with IC50 drug dose for at least 3 months. Resistant cells were maintained in drug-free medium for 2 weeks before subsequent experiments. Parental cells were cultured synchronously, unexposed to drug, as a control for all experiments (called sensitive cells).

### MTT assay

Cells were seeded in 96-well plates followed by doxorubicin or paclitaxel incubation for 72 h. Following medium removal and PBS washing, cells were incubated with 3-(4,5-dimethylthiazol-2-yl)-2,5-diphenyltetrazolium bromide (MTT solution) in six replicates per drug dose or control. Cell viability was expressed as percent compared to control group (without drug).

### Migration assay

The *in vitro* scratch assay is a suitable and valuable method to study migration capacity because mimics the behavior of cells during migration *in vivo*^47^. MCF-7 and MDA-MB-231 breast cells were co-culture with respective exosome and seeded at a cell density of 2 × 10^5^ cells/well into a 24-well plate and incubated at 37 °C under a water-saturated 95% air-5% CO_2_ atmosphere. Thereafter, cells were scraped with a P200 pipette tip, washed with PBS and the media was replaced by media without FBS. The cells were then photographed by inverted microscopy at ×5 magnification at 0 h, 24 h and 48 h.

### mRNA expression analysis

Total RNA was extracted with Trizol (Life Technologies). Reverse transcription was performed using 2 μg of RNA and the High-Capacity cDNA Reverse Transcription Kit (Life Technologies). Real-time PCR was performed on the resulting cDNA using the Power SYBR® Green Master Mix (Thermo Scientific), 10 mmol/L of each primer (Table 2) and 20 ng of cDNA in a 7500 real-time PCR system (Applied Biosystems, USA, CA). The results were analyzed using the 2^−ΔΔCt^ relative quantification method with 18 S as internal control.

**Table 2:** Primers used to mRNA expression analysis.

	Forward primer	Reverse primer
BMI1	AATCCCCACCTGATGTGTGT	GCTGGTCTCCAGGTAACGAA
SLUG	CCCTGAAGATGCATATTCGGAC	CTTCTCCCCCGTGTGAGTTCTA
SNAIL	GCTGCAGGACTCTAATCCAGAGTT	GACAGAGTCCCAGATGAGCATTG
E-CAD	TCATGAGTGTCCCCCGGTAT	CAGCCGCTTTCAGATTTTCAT
SOX9	AGCGAACGCACATCAAGAC	CTGTAGGCGATCTGTTGGGG
EZH2	GATGATGGAGACGATCCTGAA	GCTGTGCCCTTATCTGGAAA
CD44	CACGTGGAATACACCTGCAA	CGGACACCATGGACAAGTTT
CD24	TGAAGAACATGTGAGAGGTTTGAC	GAAAACTGAATCTCCATTCCACAA
ALDH1	TAAGCATCTCCTTACAGTCAC	TGTTAAGTACTTCAAGAGTCAC
TGF-β	CTGAACCCGTGTTGCTCTC	GAGGTATCGCCAGGAATTGT
C/EBP-β	CGACTTCCTCTCCGACCTC	AGGCTCACGTAGCCGTACTC
FOXO-3a	CCTTTGCCAAATCTGCTCTC	GGGCCATTAACAGTCTCTGC
18S	CGCGGTTCTATTTTGTTGGT	CGGTCCAAGAATTTCACCTC

### miR-155 expression analysis

Total RNA was reverse-transcribed using the miScript II RT Kit (QIAGEN, Hilden, Germany) following the manufacturer’s protocol. Real-time PCR was performed on the resulting cDNA using hsa-miR-155-5p specific TaqMan primer and TaqMan Universal PCR Master Mix in a 7500 real-time PCR system (Applied Biosystems). The expression of hsa-miR-423 (TaqMan primers, Applied Biosystems) was used as endogenous control.

### Exosome isolation and identification

Exosomes were harvested from culture medium of DOX-resistant, PTX-resistant, CSCs, sensitive, miR-155 mimic, and control MCF-7 cells and DOX-resistant, PTX-resistant and sensitive MDA-MB-231 cells cultured in p100 plates with DMEM with 10% exosome-depleted FBS (except for CSCs that is already cultured in absence of FBS), 100 U/ml penicillin, and 100 mg/ml streptomycin using Total Exosome Isolation Kit (Life Technologies). Briefly, the cell culture media of each condition was harvested and centrifuged at 2000 × g for 30 minutes to remove cells and debris. Then, the supernatant was mixed with 0.5 volume of Total Exosome Isolation Kit reagent and incubated at 4 ^o^C overnight. After incubation, the samples were centrifuged at 10,000 × g for 1 hour at 4 °C and the pellet containing exosome resuspended in PBS. Then, the different exosomes were used to co-culture assays and the exosomal RNA and protein were extracted using the Total Exosome RNA and Protein Isolation Kit (Life Technologies) according to the manufacturer’s protocols.

The exosomal protein concentration was measured by BCA colorimetric method and western blot analysis of exosome-related proteins CD-63 and CD-9 were performed following standard procedures. About 20 μg of the exosomal protein was separated on 8,5% SDS-PAGE gel followed by transfer to a nitrocellulose membrane (Bio-Rad, Hercules, CA, USA). After blocking with 5% skim milk powder in Tris-buffered saline (TBS)-Tween for 1 h, the membranes were incubated with specific antibody against CD-63 and CD-9 (Abcam, Cambridge, MA, USA). HRP-linked anti-rabbit secondary antibody (DAKO Corporation) was used at a 1:2000 dilution. Signal was detected by chemiluminescence using ECL substrate (Amersham Bioscience, Uppsala, Sweden).

### Exosome uptake assay

Exosomes were labeled with the red fluorescent dye PKH26 (Sigma-Aldrich) according to the manufacturer’s recommendation. Briefly, isolated exosomes from culture media were resuspended in 1 mL of Diluent C. Then, 4 μL PKH26 was diluted in another 1 mL Diluent C. The samples were mixed gently for 5 min, after which 5 mL 1% bovine serum albumin was added to bind the excess dye. The mixture containing stained exosomes was subsequently ultracentrifuged at 100,000 g for 2 h at 4^o^C, washed with PBS, resuspended in complete medium and finally incubated with sensitive cells. As the negative control, exosomes without PKH26 staining were used. Incorporation of exosomes into sensitive cells was visualized by fluorescence microscopy (Carl Zeiss, Germany) after incubation with PKH26-labeled exosomes for 18 hours at 37^o^C.

### Co-cultured assays

To investigate the potential transmission of chemoresistance, MCF-7 and MDA-MB-231 cell lines were seeded in 6-well plates and incubated with exosomes from DOX-resistant, PTX-resistant, CSCs, sensitive cells, miR-155 mimic, and miR control in DMEM with 10% exosome-depleted FBS. Following 48 hours of co-culture, one part of the cells was used for subsequently mRNA and miR-155 expression analysis and another one was used for chemoresponse analysis by MTT assay, as described previously.

### E-cadherin immunofluorescence

MCF-7 cells transfected with miR-155 or control and co-cultured with exosomes from miR-155 mimic or control were seeded on cover slips, fixed with 4% paraformaldehyde at room temperature for 10 min and then permeabilized and blocked with PBS 1X-0,3% Triton −5% FBS for 1 hour. The cells were incubated with primary antibody (anti-e-cadherin, Cell Signaling Technology, Beverly, MA, USA) at room temperature for 2 hours. After washing with PBS, cells were incubated with the secondary antibody Alexa Fluor-conjugated goat anti-mouse IgG at room temperature for 1 h. After a final wash with PBS, cover slips were mounted with anti-fading mounting medium containing 4,6-diamidino-2-phenylindole (DAPI).

### Data evaluation

The statistical analyses were conducted using SPSS 12.0 version statistical software program (SPSS, Chicago, IL). Data are presented as mean values ± S.E.M. Statistical significance (p-values) was calculated using the Student’s t test. Asterisks (* and **) indicate statistically significant differences (p < 0.05, and p < 0.01, respectively).

## Electronic supplementary material

Supplementary Figure

## References

[1] Gottesman MM (2002). Mechanisms of cancer drug resistance. Annual review of medicine.

[2] Simons M, Raposo G (2009). Exosomes–vesicular carriers for intercellular communication. Current opinion in cell biology.

[3] Lee, G., Hall, R. R., 3rd & Ahmed, A. U. Cancer Stem Cells: Cellular Plasticity, Niche, and its Clinical Relevance. *Journal of stem cell research & therapy* **6**, 10.4172/2157-7633.1000363 (2016). 10.4172/2157-7633.1000363 PMC5123595 27891292

[4] Dave B, Mittal V, Tan NM, Chang JC (2012). Epithelial-mesenchymal transition, cancer stem cells and treatment resistance. Breast cancer research: BCR.

[5] Proctor E (2013). Bmi1 enhances tumorigenicity and cancer stem cell function in pancreatic adenocarcinoma. PloS one.

[6] Xia H, Hui KM (2014). Mechanism of cancer drug resistance and the involvement of noncoding RNAs. Current medicinal chemistry.

[7] Pigati L (2010). Selective release of microRNA species from normal and malignant mammary epithelial cells. PloS one.

[8] Hannafon BN (2016). Plasma exosome microRNAs are indicative of breast cancer. Breast cancer research: BCR.

[9] Wang F (2015). The Value of MicroRNA-155 as a Prognostic Factor for Survival in Non-Small Cell Lung Cancer: A Meta-Analysis. PloS one.

[10] Shen R (2015). MiRNA-155 mediates TAM resistance by modulating SOCS6-STAT3 signalling pathway in breast cancer. American journal of translational research.

[11] Ouyang M (2014). MicroRNA profiling implies new markers of chemoresistance of triple-negative breast cancer. PloS one.

[12] Johansson J (2013). MiR-155-mediated loss of C/EBPbeta shifts the TGF-beta response from growth inhibition to epithelial-mesenchymal transition, invasion and metastasis in breast cancer. Oncogene.

[13] Kong W (2010). MicroRNA-155 regulates cell survival, growth, and chemosensitivity by targeting FOXO3a in breast cancer. The Journal of biological chemistry.

[14] O’Brien K (2013). Exosomes from triple-negative breast cancer cells can transfer phenotypic traits representing their cells of origin to secondary cells. European journal of cancer.

[15] Chen WX (2014). Exosomes from drug-resistant breast cancer cells transmit chemoresistance by a horizontal transfer of microRNAs. PloS one.

[16] Fischer KR (2015). Epithelial-to-mesenchymal transition is not required for lung metastasis but contributes to chemoresistance. Nature.

[17] Clevers H (2011). The cancer stem cell: premises, promises and challenges. Nature medicine.

[18] Liu F, Kong X, Lv L, Gao J (2015). TGF-beta1 acts through miR-155 to down-regulate TP53INP1 in promoting epithelial-mesenchymal transition and cancer stem cell phenotypes. Cancer letters.

[19] Yu DD (2015). Role of miR-155 in drug resistance of breast cancer. Tumour biology: the journal of the International Society for Oncodevelopmental Biology and Medicine.

[20] Wen SW (2016). The Biodistribution and Immune Suppressive Effects of Breast Cancer-Derived Exosomes. Cancer research.

[21] Costa-Silva B (2015). Pancreatic cancer exosomes initiate pre-metastatic niche formation in the liver. Nature cell biology.

[22] Valadi H (2007). Exosome-mediated transfer of mRNAs and microRNAs is a novel mechanism of genetic exchange between cells. Nature cell biology.

[23] Qamar S, Carrasquer CA, Cunningham SL, Cunningham AR (2011). Anticancer SAR models for MCF-7 and MDA-MB-231 breast cell lines. Anticancer research.

[24] Li W (2010). The clinicopathological significance of CD44+/CD24−/low and CD24+ tumor cells in invasive micropapillary carcinoma of the breast. Pathology, research and practice.

[25] Guttilla IK (2012). Prolonged mammosphere culture of MCF-7 cells induces an EMT and repression of the estrogen receptor by microRNAs. Breast cancer research and treatment.

[26] Kajiyama H (2007). Chemoresistance to paclitaxel induces epithelial-mesenchymal transition and enhances metastatic potential for epithelial ovarian carcinoma cells. International journal of oncology.

[27] Hiscox S (2006). Tamoxifen resistance in MCF7 cells promotes EMT-like behaviour and involves modulation of beta-catenin phosphorylation. International journal of cancer. Journal international du cancer.

[28] Chang JC (2005). Patterns of resistance and incomplete response to docetaxel by gene expression profiling in breast cancer patients. Journal of clinical oncology: official journal of the American Society of Clinical Oncology.

[29] Tiago DM, Conceicao N, Caiado H, Laize V, Cancela ML (2016). Matrix Gla protein repression by miR-155 promotes oncogenic signals in breast cancer MCF-7 cells. FEBS letters.

[30] Cheng CJ (2015). MicroRNA silencing for cancer therapy targeted to the tumour microenvironment. Nature.

[31] Chiu CF (2016). NF-kappaB-driven suppression of FOXO3a contributes to EGFR mutation-independent gefitinib resistance. Proceedings of the National Academy of Sciences of the United States of America.

[32] Chen WX (2014). MicroRNAs delivered by extracellular vesicles: an emerging resistance mechanism for breast cancer. Tumour biology: the journal of the International Society for Oncodevelopmental Biology and Medicine.

[33] Hannafon BN, Ding WQ (2013). Intercellular communication by exosome-derived microRNAs in cancer. International journal of molecular sciences.

[34] Zeng AL (2017). Tumour exosomes from cells harbouring PTPRZ1-MET fusion contribute to a malignant phenotype and temozolomide chemoresistance in glioblastoma. Oncogene.

[35] Crompot E (2017). Extracellular vesicles of bone marrow stromal cells rescue chronic lymphocytic leukemia B cells from apoptosis, enhance their migration and induce gene expression modifications. Haematologica.

[36] Bandari, S. K. *et al*. Chemotherapy induces secretion of exosomes loaded with heparanase that degrades extracellular matrix and impacts tumor and host cell behavior. *Matrix biology: journal of the International Society for Matrix Biology*, 10.1016/j.matbio.2017.09.001 (2017). 10.1016/j.matbio.2017.09.001 PMC5816689 28888912

[37] Ji R (2015). Exosomes derived from human mesenchymal stem cells confer drug resistance in gastric cancer. Cell cycle.

[38] Hu Y (2015). Fibroblast-Derived Exosomes Contribute to Chemoresistance through Priming Cancer Stem Cells in Colorectal Cancer. PloS one.

[39] Yin Q (2008). MicroRNA-155 is an Epstein-Barr virus-induced gene that modulates Epstein-Barr virus-regulated gene expression pathways. Journal of virology.

[40] Xiao B (2009). Induction of microRNA-155 during Helicobacter pylori infection and its negative regulatory role in the inflammatory response. The Journal of infectious diseases.

[41] Xie S, Chen H, Li F, Wang S, Guo J (2015). Hypoxia-induced microRNA-155 promotes fibrosis in proximal tubule cells. Molecular medicine reports.

[42] Liu F, Kong X, Lv L, Gao J (2015). MiR-155 targets TP53INP1 to regulate liver cancer stem cell acquisition and self-renewal. FEBS letters.

[43] Li DP (2017). MiR-155 up-regulated by TGF-beta promotes epithelial-mesenchymal transition, invasion and metastasis of human hepatocellular carcinoma cells *in vitro*. American journal of translational research.

[44] Mikamori M (2017). MicroRNA-155 Controls Exosome Synthesis and Promotes Gemcitabine Resistance in Pancreatic Ductal Adenocarcinoma. Scientific reports.

[45] Dontu G (2003). *In vitro propagation a*nd transcriptional profiling of human mammary stem/progenitor cells. Genes & development.

[46] Ponti D (2005). Isolation and *in vitro* propagation of tumorigenic breast cancer cells with stem/progenitor cell properties. Cancer research.

[47] Liang CC, Park AY, Guan JL (2007). *In vitro* scratch assay: a convenient and inexpensive method for analysis of cell migration *in vitro*. Nature protocols.

