# Supplementary material for: Exosome-mediated breast cancer chemoresistance via miR-155 transfer
Source: Sci Rep. 2018 Jan 16;8:829. doi: 10.1038/s41598-018-19339-5 (PMC5770414; doi:10.1038/s41598-018-19339-5)
Supplement: Supplementary file 1 — Supplementary Figure [file 41598_2018_19339_MOESM1_ESM.doc]

**Supplementary information for**

**Exosome-mediated breast cancer chemoresistance via miR-155 transfer**

Juliana Carvalho Santos^1*^; Natália Lima^2^; Luis Otavio Sarian^1^; Ander Matheu^3^; Marcelo Ribeiro^2*^ & Sophie Derchain^1^

^1^Women’s Health Hospital “Prof Dr José Aristodemo Pinotti” (CAISM), State University of Campinas (UNICAMP), Campinas, SP, Brazil. ^2^Clinical Pharmacology and Gastroenterology Unit, São Francisco University, São Francisco University, Bragança Paulista, SP, Brazil.^3^Cellular Oncology Group, Biodonostia Health Research Institute, San Sebastian, Spain.

*Correspondence

Juliana C. Santos, Ph.D

R. Alexandre Fleming, 79

Campinas - SP Zip Code 13083-881

Email: [santos.j.c@outlook.com](mailto:santos.j.c@outlook.com)

Marcelo L Ribeiro, Ph.D

Avenida São Francisco de Assis, 218

Bragança Paulista-SP, Brazil. Zip Code 12916-900

Email: [marcelo.ribeiro@usf.edu.br](mailto:marcelo.ribeiro@usf.edu.br)

This file include Supplementary Figure S1.


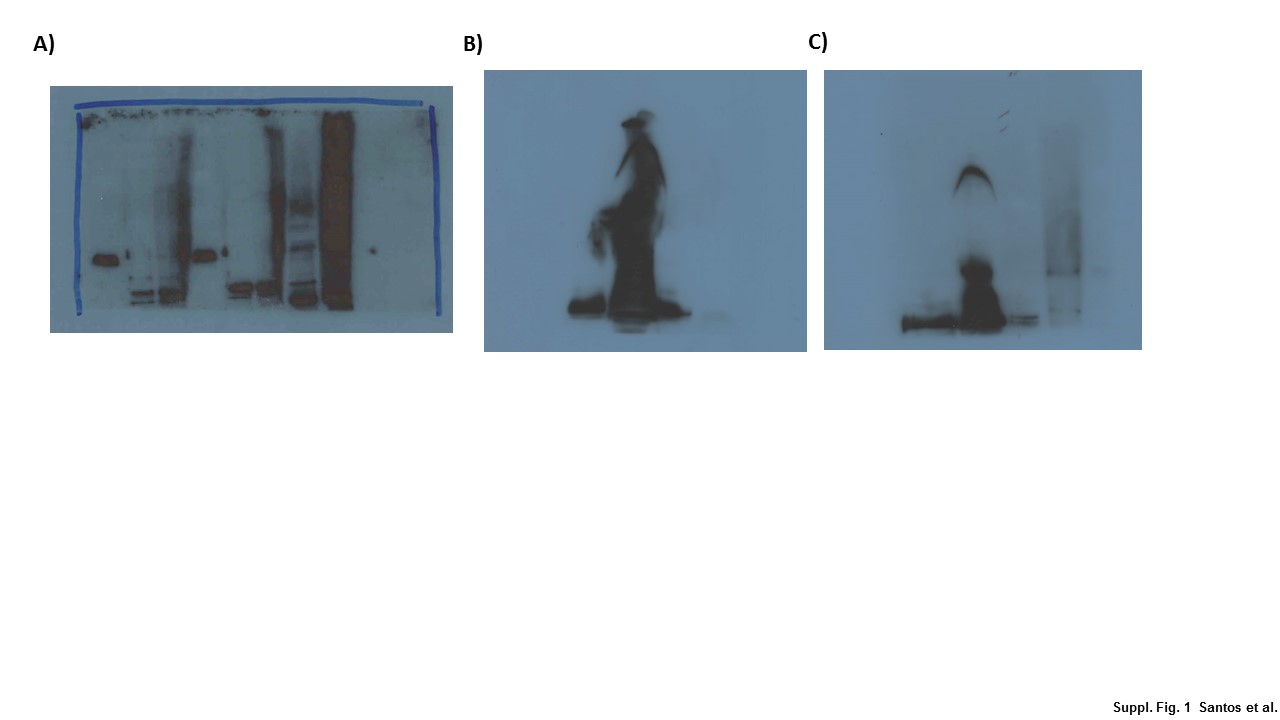
**Supplementary Figure S1 –** Full-length blots of CD-63 (A) and CD-9 (C and D) exosome-related proteins.
